# Editorial: Alternative and complementary therapies to promote mental health and wellbeing for older adults, volume II

**DOI:** 10.3389/fpsyg.2025.1583131

**Published:** 2025-04-28

**Authors:** Meiling Qi, Shu-Chuan Chen, Cindy Jones

**Affiliations:** ^1^School of Nursing and Rehabilitation, Shandong University, Jinan, China; ^2^Department of Nursing, National Tainan Junior College of Nursing, Tainan, Taiwan; ^3^Faculty of Health Sciences & Medicine, Bond University, Gold Coast, QLD, Australia

**Keywords:** alternative therapy, complementary approaches, mental health, wellbeing, older adults

As the global population ages, the mental health and wellbeing of older adults have become a crucial public health concern (Reynolds et al., 2022). Issues such as depression, anxiety, and cognitive decline not only diminish individuals’ quality of life but also place significant burdens on healthcare systems. The growing recognition of alternative and complementary therapies as effective approaches to address these challenges was highlighted in our previous editorial (Qi and Jones, 2023). This special issue features six articles exploring various therapies, including acupuncture, calligraphy, square dancing, and virtual reality interventions, offering valuable insights into their effectiveness and underlying mechanisms.

Acupuncture has demonstrated potential in addressing mental health disorders linked to neurological conditions (Yang et al., 2022). Wei et al. found that acupuncture influences EEG microstates in middle-aged and older patients with post-stroke depression (PSD), suggesting its potential as a biomarker for treatment success. The study provides a fresh perspective on the neurophysiological mechanisms of PSD, highlighting electroencephalography microstates as a functional biomarker. Additionally, two protocol studies by Xiao et al. and Shi et al. examine acupuncture’s effectiveness in alleviating depression in Parkinson’s disease patients and treating generalized anxiety disorder in young adults, respectively, using functional magnetic resonance imaging and event-related potentials in randomized controlled trials. These investigations offer insights into how acupuncture may reduce depression and anxiety levels while uncovering its neurophysiological mechanisms.

Ouyang et al. illustrate how square dancing, a social and physical activity, enhances the quality of life for middle-aged and older adults. This form of exercise not only promotes physical health but also provides psychological benefits by reducing negative emotions and fostering a positive outlook on aging. This aligns with a holistic health perspective, emphasizing the connection between physical and mental wellness. Similarly, Wang and Tang highlight how calligraphy can positively impact mental health by promoting concentration and relaxation, enhancing tranquility, and aiding in stress management. This non-pharmacological method can be seamlessly integrated into daily routines and may particularly appeal to older adults with an interest in traditional cultural practices.

Furthermore, Stasolla et al. introduce virtual reality interventions featuring digital storytelling as an innovative approach to assessing and rehabilitating patients with Alzheimer’s disease. The immersive qualities of virtual reality, combined with storytelling, stimulate cognitive functions and enhance emotional wellbeing. This method also offers potential benefits for caregivers by providing new ways to engage patients in therapeutic activities.

Despite the promising results associated with these alternative therapies, several challenges and limitations must be addressed. Many studies included in this special issue had relatively small sample sizes, potentially limiting the generalizability of their findings. For instance, Shi et al.’s study included only 28 participants per group, which may impact the reliability of the results. Sampling bias was also present, as seen in Wang and Tang’s research, where convenience sampling led to an overrepresentation of certain demographics, failing to fully represent the broader older adult population. Moreover, the mechanisms underpinning these therapies remain inadequately understood. While some studies have proposed explanations, such as acupuncture’s influence on brain networks or calligraphy’s psychological benefits, further research is needed to clarify these connections. A lack of standardization in treatment protocols also presents a challenge; variations in acupoints, stimulation techniques, and treatment durations for acupuncture make it difficult to compare results across studies.

Future research should aim to address these limitations. Large-scale randomized controlled trials with extended follow-up periods are necessary to confirm the efficacy and longevity of these treatments. More representative sampling methods should be employed to ensure findings are applicable to a broader population. Additionally, ongoing investigation into the underlying mechanisms of these therapies is essential for optimizing treatment protocols and creating targeted interventions. For instance, deeper research into acupuncture’s neural mechanisms may lead to the identification of specific acupoints and stimulation parameters tailored to different mental health conditions (Cui et al., 2021). Further exploration of interactions between alternative therapies and their combination with conventional treatments is also needed (Qi et al., 2020). For example, acupuncture combined with medication for PSD patients or virtual reality as an adjunct to cognitive rehabilitation for Alzheimer’s patients warrants further study.

The findings presented in this special issue hold significant implications for clinical practice. Healthcare professionals should familiarize themselves with alternative therapies and consider them in patient care strategies. For example, older adults experiencing mild anxiety or stress-related issues may benefit from acupuncture, calligraphy, or square dancing as part of an integrative treatment approach. However, it is equally important for healthcare providers to recognize the limitations and potential risks associated with these therapies, ensuring that patients are well-informed and actively involved in their treatment decisions. Additional training in these therapies for healthcare professionals is crucial to facilitate their effective implementation and oversight.

In conclusion, the six articles in this special issue provide valuable perspectives on how alternative and complementary therapies can enhance the mental health and wellbeing of older adults. Acupuncture, square dancing, calligraphy, and virtual reality interventions show considerable promise in addressing various mental health challenges in this population. Nonetheless, further research is needed to overcome existing limitations. Continued exploration of these therapies will contribute to the development of more effective and holistic strategies for supporting the mental health of older adults and promoting healthy aging. Future research should prioritize large-scale trials, studies focused on underlying mechanisms, and assessments of the long-term effects of these interventions to enhance the care and wellbeing of older adults.
